# Discrepant End-Tidal Concentrations of Sevoflurane at the Same A-Line Autoregressive Index Level during Induction of General Anesthesia: An Observational Study

**DOI:** 10.3390/jcm10194526

**Published:** 2021-09-30

**Authors:** Che-Hao Hsu, Shung-Tai Ho, Chih-Cherng Lu, Ju-O Wang, Te-Chun Yeh, Tso-Chou Lin

**Affiliations:** 1 Department of Anesthesiology, Tungs’ Taichung MetroHarbor Hospital, Taichung 43503, Taiwan; hsuchehao@yahoo.com.tw; 2 Department of Anesthesiology, Tri-Service General Hospital, National Defense Medical Center, Taipei 11490, Taiwan; shungtai0617@gmail.com; 3 Department of Anesthesiology, Kaohsiung Medical University Hospital, Kaohsiung Medical University, Kaohsiung 80756, Taiwan; 4 Department of Anesthesiology, Taipei Veterans General Hospital, National Defense Medical Center, Taipei 11217, Taiwan; chihchernglu510803@gmail.com; 5 School of Public Health, National Defense Medical Center, Taipei 11490, Taiwan; carolwamg@gmail.com; 6 Development and Planning Center, Taipei City Hospital, Taipei 10341, Taiwan; chun0417@gmail.com

**Keywords:** A-line Autoregressive Index (AAI), end-tidal concentration, general anesthesia, induction, sevoflurane

## Abstract

Background: The A-Line Autoregressive Index (AAI), which is derived from auditory evoked potentials, has been used for determining anesthetic depth. This study verified the correlation between AAI values and the corresponding end-tidal concentrations of sevoflurane during general anesthesia induction. Methods: Thirty young male adults undergoing elective minor orthopedic surgery were sequentially allocated to receive inspiratory 3%, 5%, or 6% sevoflurane for mask induction, followed by mechanical ventilation after tracheal intubation. The inspiratory, end-tidal and estimated jugular bulb concentrations of sevoflurane were recorded at three target AAI values: below 20, below 10, and at the start of burst suppression. Results: The mean time to loss of consciousness in the 6% sevoflurane group was shorter than that in the 5% and 3% groups; however, the groups had comparable AAI values (range: 16–45). The 6% group had a higher end-tidal concentration (4.5% ± 0.2% vs. 3.8% ± 0.2%, *p* < 0.05) than did the 5% group, despite having the same target anesthetic levels by AAI score ≤10, whereas the estimated jugular bulb concentrations were comparable (1.9% vs. 1.9%) in both groups. Conclusions: Following mechanical ventilation with inspiratory 3%, 5%, or 6% sevoflurane, the end-tidal concentrations were discrepant at the same end points of AAI levels, despite similar estimated jugular bulb concentrations of sevoflurane. Thus, conventional alveolar concentration may overestimate anesthesia depth during rapid wash-in of sevoflurane.

## 1. Introduction

Use of the A-Line Autoregressive Index (AAI) by autoregressive modelling with exogenous input (ARX-model) enables quicker extraction of middle-latency auditory evoked potentials [1]. In the past two decades, the AAI has been applied to differentiate degrees of anesthetic effects [2], including the correlation with the end-tidal concentrations of sevoflurane [3] and desflurane [4]. The predictable and dose-dependent response in a graded, reversible manner to minimal alveolar concentrations of volatile anesthetics provides a window to the brain for the assessment of matters such as potential awareness, burst suppression [2], and adjustment of anesthesia depth [5].

For inhalational anesthetics, however, a time lag between end-tidal and brain concentrations has been identified and physiologically modelled through measurement of the inspiratory, end-tidal, and jugular bulb concentrations [6,7,8]. Accordingly, the body’s uptake and elimination [9] of anesthetics across the alveolar membrane and blood–brain barrier depend on the partition coefficient [10,11,12], ventilation [13], and cardiac output [7]. Sevoflurane requires nearly 40 min to achieve a pharmacokinetic equilibrium between arterial and jugular bulb concentrations, indicating that no further brain uptake of inspiratory 3.5% sevoflurane occurs after general anesthesia induction [7].

Clinically, using a higher inspiratory concentration of anesthetic could allow practitioners to adjust and achieve the target anesthetic level more rapidly and obtain a higher end-tidal concentration. During the wash-in period of anesthesia, data regarding the time delay between end-tidal concentrations [14] and their electroencephalographic effects, as revealed by the modern AAI, are scarce [5]. Therefore, this study examined the clinical correlations between AAI values following mechanical ventilation with different inspiratory concentrations of sevoflurane and their corresponding inspiratory and end-tidal (alveolar) concentrations during induction of general anesthesia.

## 2. Materials and Methods

### 2.1. Participants

After obtaining approval from the relevant institutional review board (TSGHIRB-096-05-0082) and informed consent from each patient, the current study enrolled male patients classified as American Society of Anesthesiologists physical status I who were undergoing elective minor orthopedic surgery. Those with coexisting obesity or hypertension were excluded.

### 2.2. Anesthetic Procedures

Thirty non-premedicated young male patients were sequentially allocated to three groups: inspiratory 3%, 5%, or 6% sevoflurane. Before mask ventilation, the anesthetic circuit was prewashed for 3 min with 3%, 5%, or 6% sevoflurane in 6 L/min oxygen. Under standard monitoring, the patients in each group were encouraged to take a deep breath of sevoflurane through an ordinary face mask, followed by normal spontaneous breathing or assisted mask ventilation for 5 min. After loss of consciousness (no response to verbal command), rocuronium (0.6 mg/kg) was administered for oral tracheal intubation. Next, mechanical normoventilation (tidal volume 6–8 mL/kg and respiratory rate 10–16/min to keep end-tidal CO_2_ 35–45 mmHg) was initiated with inspiratory 3%, 5%, or 6% sevoflurane in 6 L/min oxygen for a maximum of 60 min or until the target sedation levels were achieved before initiating surgery. Hemodynamic variables, including heart rate, blood pressure, pulse oximetry, and capnography, were recorded at the start of induction and at 5, 10, 15, 20, 30, 40, 50, and 60 min, and inspiratory and end-tidal concentrations of sevoflurane were measured using side-stream gas sampling with a CardioCap 5 monitor (Datex, Helsinki, Finland). Intravenous bolus of nitroglycerin (50–100 mcg) for maintaining a systolic blood pressure below 160 mmHg during tracheal intubation and ephedrine (5–10 mg) for maintaining a subsequent mean blood pressure above 60 mmHg were incrementally administered, as required. No other intravenous medications were administered during the study period.

### 2.3. Data Acquisition

An auditory evoked potential (AEP) monitor (AEP Monitor/2; version 1.6; Danmeter A/S, Odense, Denmark) was applied to the patients’ forehead with contact impedance < 5 K Ohm. Headphones connected to the device provided auditory stimulus, an intermittent click (6 Hz in frequency with automatically controlled volume according to the appropriate AEP extraction quality conditions). Processing time for the AAI was 30 s for the first detected signal, with a total update delay of 6 s. The awake AAI level before anesthesia was reset to a maximal 60 value for all patients. An AAI range of 0–60 is recommended to minimize oscillations while the patient is awake (high AAI values) and to provide higher-quality graphical resolutions when the patient is asleep or anesthetised (low AAI values) [15]. The monitor included a burst suppression indicator to indicate periods when the electroencephalogram (EEG) was isoelectric. The AEP monitor indicated the percentage of burst suppression over the preceding 30 s of the EEG signal. After loss of consciousness, the aforementioned hemodynamic and inhalation parameters were also collected at three target AAI levels: below 20, below 10, and at the beginning of burst suppression. The estimated jugular bulb concentrations of sevoflurane were calculated using the end-tidal concentrations in this study and the calculated ratios of jugular bulb over end-tidal concentrations from a related pharmacokinetic study of sevoflurane brain uptake [7]. The ratios were 0.378 at 5 min, 0.461 at 10 min, 0.503 at 20 min, 0.550 at 30 min, and 0.635 at 60 min following mechanical ventilation.

### 2.4. Data Analysis

Statistical analysis was conducted using SPSS version 22 (IBM Corp., Armonk, NY, USA). Descriptive statistical analyses were performed to calculate the means and standard deviations of demographic and clinical variables. Since the data did not satisfy the assumptions of equality of variance and normal distribution, the Kruskal–Wallis test and Mann–Whitney U-test were employed to analyse the change in values for intergroup comparison. When a significant difference was observed between the groups, Dunn’s test was performed to identify the deviating group. Differences with *p* < 0.05 were considered statistically significant.

## 3. Results

### 3.1. Participant Recruitment

Thirty adult male patients undergoing elective minor orthopedic surgery were sequentially allocated to three groups of inspiratory 3%, 5%, or 6% sevoflurane for induction of general anesthesia.

### 3.2. General Characteristics of Participants

The general data are presented in Table 1. The 6% sevoflurane group had a shorter time to loss of consciousness but similar AAI values relative to the 5% and 3% groups. The awake AAI level before anesthesia was reset to 60 for each patient.

### 3.3. Iinspiratory and End-Tidal Concentrations of Sevoflurane

**Figure 1:**
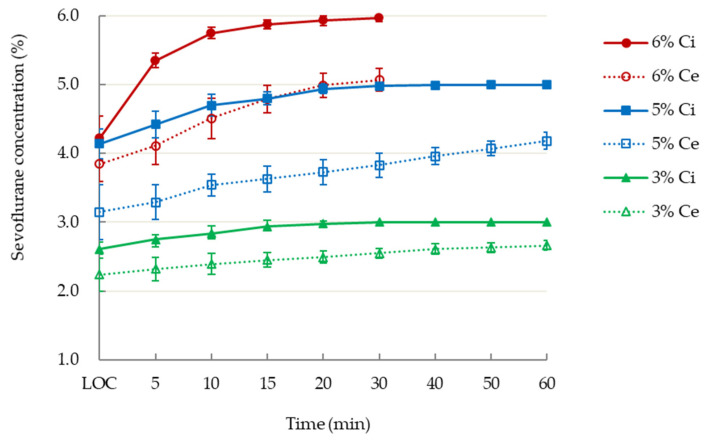
T Inspiratory (Ci) and end-tidal (Ce) concentrations of inspiratory 3%, 5%, or 6% sevoflurane following loss of consciousness (LOC) during administration of general anesthesia.

Figure 1 illustrates the pharmacokinetics of inspiratory and end-tidal concentrations of sevoflurane in the three groups. The administration of sevoflurane in the 6% group was terminated at 30 min after burst suppression status had been achieved.

### 3.4. AAI Values and Sevoflurane Concentrations

The time required to reach the programmed AAI levels and their corresponding inspiratory, end-tidal and estimated jugular bulb concentrations are summarised in Table 2. The AAI scores in the 3% sevoflurane group did not fall below 10 during the 60-min period (14.6 ± 2.3 at 60 min). The 6% sevoflurane group were terminated at 30 min on account of low AAI scores (0.9 ± 2.8) and burst suppression. At the same AAI end points, including values ≤ 20, values ≤ 10, and at the start of burst suppression, the mean end-tidal concentrations in the 6% sevoflurane group were significantly higher than those in the 5% sevoflurane group, whereas the estimated jugular bulb concentrations were comparable between these two groups. The estimated jugular bulb concentration at 30 min in the 6% group was twice that in the 3% group (2.8% ± 0.1% vs. 1.4% ± 0.1%, respectively).

### 3.5. Hemodynamic Variables during Induction

Table 3 provides the comparable hemodynamic variables. Three patients in the 5% group and three patients in the 6% group received intermittent ephedrine 5 mg (max 15 mg in total) to maintain their mean arterial blood pressure above 60 mmHg. None of the patients experienced adverse events.

## 4. Discussion

### 4.1. Main Findings

By using AAI monitoring, we demonstrated that, relative to patients receiving inspiratory 5% or 3% sevoflurane, those receiving inspiratory 6% sevoflurane had a shorter time to loss of consciousness with comparable AAI values and a shorter time required to reach the target AAI values under mechanical ventilation but higher end-tidal concentrations at the same end-point AAI values. The estimated jugular bulb concentrations were similar between the 6% and 5% groups at the same AAI levels. These findings indicate that alveolar concentration may overestimate anesthesia depth because of a notable gap between end-tidal and brain concentrations during rapid wash-in of a higher concentration of sevoflurane.

### 4.2. Higher Inspiratory and End-Tidal Concentrations at the Same AAI Values during Wash-In

The minimum alveolar concentration (MAC) required to prevent movement in response to surgical incision in 50% of patients has been used as an indicator of anesthesia depth of inhalational anesthetics for decades [16]. This value is derived from a steady end-tidal concentration representing the arterial and brain concentrations of the anesthesia. Lu et al. [7] demonstrated that the ratio of arterial/end-tidal concentration of sevoflurane remained at 0.63 after 30 min of mechanical ventilation with inspiratory 3.5% sevoflurane. They also revealed that a higher inspiratory concentration of isoflurane could accelerate its brain uptake [8]. Equilibrium between arterial and jugular bulb concentrations was achieved after 40 min for inspiratory 2% isoflurane and 50 min for 1% isoflurane [8]. The arterial and jugular bulb concentrations in the 2% isoflurane group were approximately double those of the 1% isoflurane group [8]. In the current study, a similar pharmacokinetic phenomenon was observed in estimated jugular bulb concentrations at 30 min (2.8% vs. 1.4%) between the 6% and 3% sevoflurane groups. As expected, the time to loss of consciousness and the time required to achieving the target AAI values were shorter in the 6% sevoflurane group. Theoretically, end-tidal concentrations for calculating MAC values as anesthesia depth should be similar at the same target AAI levels regardless of whether inspiratory 5% or 6% sevoflurane is used. However, the actual end-tidal concentrations were higher in the 6% group at the same AAI values: ≤20, ≤10, and at the start of burst suppression. The estimated jugular bulb concentrations were similar at the same end points of AAI values between the 6% and 5% sevoflurane groups, which is consistent with our previous observations regarding the pharmacokinetics of sevoflurane uptake [7]. This finding indicates a lag in the time required to achieve transition of anesthesia across the alveolar membrane and blood–brain barrier, [17] which is determined by various blood/gas and brain/blood partition coefficients of inhalational anesthetics [18]. The MAC value might be overestimated as a result of higher end-tidal concentration during wash-in with a higher inspiratory concentration of anesthetics, which may result in de facto inadequate anesthesia depth under surgical stimulation.

### 4.3. EEG Processing for Adjustment of Anesthesia Depth

A composite AEP index incorporating EEG has been used as an indicator for anesthesia depth according to dose [2] and in relation to age, [3] which is a more discriminant predictor of different clinical states of general anesthesia [19]. However, some studies have failed to observe a graded response with steady-state end-tidal concentrations of sevoflurane, neither decreasing from 2% to 1.5% and 1% in adults [20] nor increasing from 1.5% to 2% and 2.5% in infants and children [21]. The so-called steady-state is based on the constant administration of an end-tidal concentration of sevoflurane for 11 min [20] and was calculated using the Gas Man Anesthesia Simulator programme for equilibration of partial pressures between the brain and the lungs [22]. Our previous pharmacokinetic study, which employed blood sampling for sevoflurane concentration analysis, demonstrated that the time required to achieve equilibration between arterial and jugular bulb concentrations (no further brain uptake) was 38.5 min following mechanical ventilation with inspiratory 3.5% sevoflurane, and a near constant end-tidal concentration was achieved after 30 min of ventilation [7]. Therefore, the early change in end-tidal concentrations during wash-in or wash-out may not accurately reflect the true brain uptake and anesthesia depth. The application of EEG processing could facilitate the adjustment of anesthesia depth during the wash-in or wash-out periods.

### 4.4. AAI Values and MAC of Sevoflurane

The AAI values in the 3% sevoflurane group did not decrease to less than 10 during 60 min of ventilation despite the ultimate end-tidal concentration being 2.7%. Young male patients were recruited for this study to reduce the impact of interindividual comorbidities and age or sex-related variability on MAC values [23]. One MAC of sevoflurane is 1.8% at the age of 40 years, with an approximate decrease of 6% every decade [23]. Women appear to have the same MAC as men [24]. However, AAI values were reported to be severely attenuated or reach a value of 0 under a 2% steady-state end-tidal concentration of sevoflurane in women aged 20–60 years [25].

### 4.5. Limitations

Two limitations of the current study should be addressed. First, all 30 patients were young male patients aged 20–25 years. The small sample size and the inclusion of only young men in this observational study may have limited the clinical application of the findings; future research could include a more diverse sample. Second, AAI values only indicated the sedation levels before surgery in our patients and not the clinical anesthesia depth during surgery. Possible confounding factors that could interfere with EEG processing were excluded, such as surgical stimulation, noisy environment, hypoglycemia, cerebral ischemia and neurological disorders, [1] and intravenous benzodiazepine and propofol.

## 5. Conclusions

We demonstrated that patients receiving a higher sevoflurane concentration had a shorter time to loss of consciousness with comparable AAI values and had higher end-tidal concentrations of sevoflurane at the same end-point AAI levels. Their estimated jugular bulb concentrations were also similar at the same AAI levels. Anesthesia depth, calculated according to alveolar concentration, may be overestimated during the rapid wash-in of inhalation anesthesia. This discrepancy between alveolar and brain concentrations at the same AAI values of anesthesia should be considered by practitioners.

## Figures and Tables

**Table 1:** Demographic data.

	Sevoflurane 3%	Sevoflurane 5%	Sevoflurane 6%	*p* Value
Patient number	10 males	10 males	10 males	
Age, year	21.9 ± 1.2	21.7 ± 1.8	21.9 ± 1.4	0.956
Height, cm	175.3 ± 8.4	169.9 ± 2.7	171.3 ± 4.7	0.293
Weight, kg	71.2 ± 10.8	64.8 ± 12.1	62.8 ± 9.3	0.206
Body mass index, kg/m^2^	23.2 ± 3.7	22.4 ± 4.1	21.3 ± 2.0	0.42
Time to	122.5 ± 35.0	89.5 ± 22.5	71.5 ± 12.5	0.002
loss of consciousness, sec	(75–180)	(55–120)	(55–90)	
AAI values at	31.7 ± 9.9	26.2 ± 7.0	28.6 ± 6.8	0.355
loss of consciousness	(16–45)	(20-36)	(20–41)	

Data are presented as the mean ± standard deviation (range). AAI, A-Line Autoregressive Index.

**Table 2:** Time required to achieve the target A-Line Autoregressive Index (AAI) values and the corresponding inspiratory, end-tidal and estimated jugular bulb concentrations of sevoflurane.

Anesthesia Level	Sevoflurane Group	Time after Induction	Inspiratory Concentration (%)	End-Tidal Concentration (%)	Estimated Jugular Bulb Concentration (%)
Loss of consciousness	3%	122.5 ± 35.0 s	2.6 ± 0.1	2.2 ± 0.2	
	5%	89.5 ± 22.5 s	4.1 ± 0.2	3.2 ± 0.4	
	6%	71.5 ± 12.5 s	4.2 ± 0.3	3.9 ± 0.3	
AAI ≤ 20	3%	5.9 ± 1.5 min	2.8 ± 0.1	2.4 ± 0.1	0.9 ± 0.0
	5%	4.8 ± 3.0 min	4.3 ± 0.2	3.4 ± 0.2	1.3 ± 0.1
	6%	2.6 ± 2.0 min	4.8 ± 0.6	4.1 ± 0.2	1.4 ± 0.1
AAI ≤ 10	5%	21.4 ± 9.8 min	4.9 ± 0.1	3.8 ± 0.2	1.9 ± 0.1
	6%	8.3 ± 2.9 min	5.6 ± 0.2	4.5 ± 0.2 *	1.9 ± 0.1
Start of burst suppression	5%	26.8 ± 11.1 min	4.9 ± 0.1	3.9 ± 0.1	2.1 ± 0.1
	6%	9.9 ± 2.6 min	5.7 ± 0.1	4.7 ± 0.1 ^#^	2.2 ± 0.1
At 30 min	3%	30 min	3.0 ± 0.0	2.6 ± 0.1	1.4 ± 0.0
	5%		5.0 ± 0.0	3.8 ± 0.2	2.1 ± 0.1
	6%		6.0 ± 0.0	5.1 ± 0.2	2.8 ± 0.1
At 60 min	3%	60 min	3.0 ± 0.0	2.7 ± 0.1	1.7 ± 0.0
	5%		5.0 ± 0.0	4.2 ± 0.1	2.7 ± 0.1

Data are presented as the mean ± standard deviation. AAI, A-Line Autoregressive Index. The estimated jugular bulb concentrations of sevoflurane were calculated using the ratios of jugular bulb over end-tidal concentrations in the previous pharmacokinetic study of sevoflurane uptake during induction [7]. * and *^#^*, *p* value < 0.001, as compared with the end-tidal concentrations in the 5% group.

**Table 3:** Hemodynamic variables.

Group	Time	Heart Rate (beats per min)	Mean Blood Pressure (mmHg)	End-Tidal CO_2_ (mmHg)
3% sevoflurane	Before induction	65.2 ± 8.7	86.9 ± 7.0	--
	Loss of consciousness	64.7 ± 7.1	82.6 ± 8.2	40.4 ± 2.7
	5 min	87.3 ± 22.2	89.1 ± 16.5	41.7 ± 3.1
	10 min	71.4 ± 16.7	73.4 ± 12.6	42.3 ± 2.0
	15 min	69.1 ± 13.8	71.6 ± 8.6	42.7 ± 1.8
	20 min	66.1 ± 12.5	69.5 ± 8.8	43.0 ± 1.1
	30 min	66.6 ± 11.8	68.9 ± 10.5	43.2 ± 1.4
	40 min	66.6 ± 12.9	67.7 ± 10.4	43.4 ± 1.3
	50 min	65.1 ±12.4	66.4 ± 8.5	43.9 ± 1.4
	60 min	63.2 ± 12.4	65.9 ± 8.6	44.1 ± 2.0
5% sevoflurane	Before induction	72.9 ± 11.7	86.3 ± 9.9	--
	Loss of consciousness	69.1 ± 11.7	81.0 ± 10.4	35.0 ± 2.6
	5 min	92.0 ± 20.4	79.7 ± 8.5	35.2 ± 2.7
	10 min	82.7 ± 14.7	74.4 ± 8.8	35.6 ± 3.1
	15 min	82.0 ± 17.6	70.6 ± 6.9	36.1 ± 3.4
	20 min	79.0 ± 16.3	69.4 ± 6.0	37.6 ± 4.0
	30 min	83.4 ± 20.0	69.6 ± 10.0	38.7 ± 4.5
	40 min	83.7 ± 21.6	65.2 ± 3.3	39.4 ± 5.1
	50 min	78.0 ± 20.4	63.0 ± 2.8	40.0 ± 5.2
	60 min	73.4 ± 17.5	61.1 ± 2.8	41.5 ± 5.7
6% sevoflurane	Before induction	72.7 ± 14.7	86.8 ± 10.6	--
	Loss of consciousness	75.1 ± 22.4	80.6 ± 9.4	38.3 ± 3.4
	5 min	93.6 ± 22.7	75.3 ± 13.9	36.7 ± 2.9
	10 min	80.6 ± 14.0	69.4 ± 13.1	35.9 ± 2.0
	15 min	81.4 ± 9.4	65.4 ± 5.0	36.1 ± 1.4
	20 min	80.3 ± 12.0	62.8 ± 4.1	36.8 ± 2.3
	30 min	79.4 ± 13.8	63.3 ± 6.5	38.0 ± 2.2

Data are presented as the mean ± standard deviation.

## Data Availability

The data presented in this study are available on request from the corresponding author.
